# Feature Extraction from Indirect Monitoring in Marine Oil Separation Systems

**DOI:** 10.3390/s18093159

**Published:** 2018-09-19

**Authors:** Graciliano Nicolás Marichal, Deivis Ávila, Ángela Hernández, Isidro Padrón, Cristina Castejón

**Affiliations:** 1 Escuela Politécnica Superior de Ingeniería, Universidad de La Laguna, 38001 Tenerife, Spain; nicomar@ull.edu.es (G.N.M.); davilapr@ull.edu.es (D.Á.); ipadron@ull.es (I.P.); 2 MAQLAB Group, Mechanical Department, Universidad Carlos III, Av. de la Universidad, 30, 28911 Madrid, Spain; castejon@ing.uc3m.es

**Keywords:** vibration, oil separator, Genetic Neuro-Fuzzy, signal processing

## Abstract

In this article, a study of characteristic vibrations of marine oils separation system is presented. Vibrations analysis allows for the extraction of representative features that could be related to the lifetime of their pieces. Actual measurements were carried out on these systems on Ro-Pax vessels to transport passengers and freight. The vibrations obtained were processed in the frequency domain and following this, they were used in a Genetic Neuro-Fuzzy System in order to design new predictive maintenance strategies. The obtained results show that these techniques as a promising strategy can be utilized to determine incipient faults.

## 1. Introduction

The maintenance of machinery implies an important investment in any industry, but it is crucial for merchant marine services. The characteristic conditions of this industry make any damage or failure occurring during a journey a dangerous situation for the crew and for the vessel itself. Owing to this, the maintenance is a fundamental aspect taken into account by the ship-owner. Usually two kind of maintenance procedures are applied: the preventive one, which is proposed by manufactures of its mechanical, electrical or electronic systems; and the corrective one, which takes place in case of failure. Nowadays, the most useful and efficient technique is predictive maintenance, and in the last years it has being used in many of industries [1,2,3], including the maritime industry [4,5,6]. This method is based on the fact that any machinery will show a signal before of failing. Therefore, knowing the normal state of machinery techniques, but by monitoring could help to prevent a breakdown. There are several predictive maintenance one of the most interesting one is which based on vibration analysis. It has been proven that out of all the non-destructive tests that can be carried out on a machine, the signature of vibrations provides the greatest amount of information about its internal functioning [1,7]. However, it is essential to design new techniques in order to conveniently extract this amount of information.

Its effectiveness has been proven over the last few years, and an example are the aircraft carriers of the US Navy, which has been using a predictive maintenance program based on vibration since 1975, with satisfactory technical and economical results [1]. With this method, vibrations are not treated as a problem, but they are a symptom. That is, vibrations will be an indicator of a problem; therefore, they will allow us to take measures to prevent failures or break downs. Overall, this is very important when dealing with critical systems, in order to avoid serious failures.

Today, monitoring of mechanical processes remains a current technical and industrial challenge. Indirect monitoring methods are less accurate, but more suitable for practical applications. Auxiliary quantities are measured and empirically correlated with machining phenomena [8].

According to on board mechanics, the separation systems or centrifuges for the cleaning of fuel and lube oils for diesel engines are considered to be complex mechanical systems as much for their functioning as for their reparation. These on board systems have little indirect monitoring over the mechanical process. Therefore, it is difficult to diagnose the state of equipment at a certain level of precision. Unfortunately, it could result in producing serious and expensive damage. The on board separators are monitored by only a speed sensor, and optionally, some systems may have an imbalance sensor and an interlocking switch [9]. However, this is too poor to detect incipient failures that could produce serious damages in a more advanced phase of the failure. Thus, it is necessary to incorporate new sensors that can measure vibrations, acoustic emissions, or electric currents of motors among others, along with adequate algorithms to detect different functioning conditions.

An on board indirect monitoring systems would be very useful to preemptively maintain vessels and thus to prevent faults and damage in the separation systems. This paper presents a condition-based maintenance technique that by intelligent algorithm, intends to process the measured signals and provides information about the internal state of the separation systems. Nowadays, the maintenance of these devices is based on number of working hours that they have been running. This means that a preventive scheduled maintenance is carried out when a certain number has been achieved. However, if there was intelligent condition monitoring, it could be possible to extend the next maintenance service if the system is healthy or make it scheduled ahead of time if the separator system shows failure indications. Therefore, in this work, a study of indirect monitoring measurements based on vibrations has been done in order to obtain the vibration signature associated with their internal state. In Section 2, a revision of related works is presented. In Section 3, a description of the Separation System used in the actual experimental tests is shown. In Section 4, an explanation of the measurement procedure is presented. In addition, signal processing methods based on artificial intelligence techniques are exposed in Section 5, Section 6 shows the results and discussions and finally a Conclusion Section (Section 7) is presented.

## 2. Related Works

Fault diagnosis has been widely investigated in different fields, and with the application of artificial intelligence, techniques have achieved improvement performance over conventional approaches. The most popular AI (Artificial Intelligence) techniques for machine diagnosis are artificial neural networks and evolutionary algorithms, particularly, genetic algorithms.

A general view can be found in [10]. An analytical approach of fault diagnosis and identification with examples of application of these techniques as model-based fault diagnosis in dynamic systems using identification techniques are presented.

In [11], an example of AI technique for fault detection is seen. In this paper, Samhouri et al. show an adaptive neuro-fuzzy inference system (ANFIS), and a neural network system (NN) for monitoring and predicting faults. In this paper, a signal related to machine condition and fault type is used, and some signal features are used as inputs to both ANFIS and neural nets, which in turn output a value for the predicted fault type.

In more recent works it can be checked that there is a tendency for the use of genetic algorithms. Chen et al. in [12] propose a fault diagnosis method for rolling bearings based on the integration of resonance-based sparse signal decomposition (RSSD) and wavelet transform (WT). Gou et al., in their work [13], are focused on detecting failures of in electric motors. Cerrada et al. in [14] expose a multi-stage feature selection mechanism for selecting the best set of condition parameters based on the time, frequency and time-frequency domains from vibration signals for fault diagnosis in gearboxes. He et al. [15] present a method based on a deep belief network (DBN) for the unsupervised fault diagnosis of a gear transmission chain. However, all of them propose a genetic algorithm in order to optimize the fault classification after data preprocessing, or to optimize the structural parameters of the training network.

From a search of improvements in maintenance policy through fault diagnosis, it has turned out that research for the optimization or the prevention of scheduled maintenance exist, but overall the field of predictive maintenance which has become the most promising. In fact, El-Thalgi et al. [16] present a review of the Predictive Health Monitoring methods and explore their capabilities, advantages, and disadvantage, particularly, in monitoring of rolling element bearings.

In the field of preventive maintenance, studies also present evolutionary algorithms. Wang et al. [17] developed a condition-based replacement and spare provisioning policy via a simulation model and a genetic algorithm. The proposed simulation model characterizes the operation, and a genetic algorithm optimizes the replacement, inspection, and inventory decision variables. In [18], Cavory et al. present a method for optimizing the schedule of maintenance tasks of all the machines in a single product manufacturing production line. They present a simulator of the production line, with a systematic approach inspired by the Taguchi method to look for the best combination of parameters for a genetic algorithm. Samrout et al. [19] uses the ant colony optimization technique based on genetic algorithms, and Lapa et al. [20] includes relevant features such as: the probability of needing a repair, the cost of such repair, typical outage times, preventive maintenance costs, etc., in their proposal of an optimization technique for preventive maintenance policies.

Finally, from state-of-the-art predictive maintenance, of Wu et al. [21] is noted. They develop an integrated neural-network-based decision support system for the predictive maintenance of rolling element bearings. This group of researchers worked with a database obtained for vibration-based degradation, and this was used in an artificial neural network model in order to estimate the life percentile and failure times of roller bearings. Finally, a cost matrix and probabilistic replacement model optimized the expected cost per unit time. Another example is [2], where a cloud-based paradigm of predictive maintenance based on mobile agent is presented. They develop a low-cost cloud sensing and computing node for sharing the analysis algorithms, and a computing node to locally process data. Also, Kaiser and Gebraeel in their paper [22] present a sensory-updated degradation-based predictive maintenance policy. The proposal is based on contemporary degradation models that combine component-specific real-time degradation signals, acquired during operation, with degradation and reliability characteristics of the component’s population to predict and update the residual life distribution (RLD). By capturing the latest degradation state of the component being monitored, the updating process provides a more accurate indication of the remaining life. With the aid of a stopping rule, maintenance routines are scheduled based on the most recently updated RLD.

Once this literature revision has been done, it seems that many researchers have decided that predictive maintenance should be accompanied by AI techniques. Our proposal is to combine predictive maintenance based on condition monitoring, with a genetic neuro-fuzzy system. However, although some many approaches related to AI have been proposed, in this case a fault detection system based on AI techniques has been shown, where it is possible to express the trained system as a set of Fuzzy rules. It is important to highlight that this characteristics of the system are desirable, due to the fact that it allows for a deeper study on the relation between signals and faults to be performed. Because of this, it is necessary to use poorly processed signals as inputs to the system. In this sense, only fast Fourier transform (FFT) processing has been chosen in this case. In the following sections, a more detailed description of our approach is exposed.

## 3. Marine Separator Systems

The separation systems are designed for cleaning lube oils and fuel for diesel engines, and for cleaning fuel oil for gas turbine engines, in marine and power applications. In this work, real measurements were carried out over the Alfa Laval S separators model [8]. This system allows for the combination of heavy fuel oil and lubricating oil treatment into a single separator.

The oil is heated and taken to the separator. After centrifugal separation, the clean oil is pumped away; meanwhile, separated impurities are accumulated at the bowl border. In the clean oil outlet, the water content is measured, and depending on its value, the control unit will open the drain valve or discard the water. The accumulated sludge is discharged to a waste tank that will be processed in the port. A more detailed description of the separation systems is given in [23].

In many cases, the centrifuges stop working due to excessive vibrations, and at that moment, it is too late to prevent or reduce the damage in the system. The most extreme vibration in the separator could be caused by: a bent bowl spindle, damaged or worn out bearings, the spindle top bearing, a broken spring, a damaged or broken belt transmission, etc. [23].

### 3.1. Alarm Function System Display

The centrifuges have an alarm system in order to guarantee a safe process. All alarms are shown on the display panel, which has easy accesses for the operators, and most of them are complemented by light emitting diodes (LEDs). There are many different alarms that can appear in the display, such as: failure in the separator motor, error in the bowl speed sensor, sludge in bowl, high sludge tank level, error in the water pressure sensor, failure in the oil pressure sensor, heater failure, high water proportion in oil, power failure, high vibration, and many others [23]. All these conditions are monitored through a direct monitoring method. At this point, it is necessary to specify the case of the vibration alarm. The oil separator system can have a vibration switch for tripping a relative increase in vibration, but it is optional. The vibration switch is sensitive to vibration in a direction that is perpendicular to its base. It contains a vibration-detecting mechanism that actuates a snap-action switch when the selected level of vibration is exceeded. This means that this device does not serve to provide a measurement of vibrations, but only as a protection mechanism, since it is not a vibration sensor. For this reason, in this work, a vibration monitoring through a sensors method is presented.

### 3.2. Abnormalities Not Displayed in the System

There are some abnormalities in the separator systems that will not be shown on the display, such as noise and smell, for example. Noise can happen for different causes, such as worn out or damaged bearings, an incorrect height position of paring disc, or an incorrect or worn out bowl assembly. An unusual smell produced by the system usually occurs during the starting process, while the friction blocks are slipping. Another possibility, and more dangerous for the system, is that the oil level in the sump may be too low. Other possible problem not displayed in the system is unsatisfactory separation; the causes of these anomalies can be caused by a wrong separation temperature, the sludge space in the bowl being full, the bowl speed being too low, the disc stack being blocked, etc.

These irregularities have many solutions; the most simple of all is to check the oil level and to add oil if necessary. Other more complex solutions are: stopping the separator system, adjusting the system, renovating the defective bearings, cleaning the disc stack, examining the electrical motor and the power transmission, checking the belt and the coupling pads, etc. [23].

### 3.3. Maintenance

Preventive maintenance is based on planned machine outage calendars, which are worked out following the recommendations of the manufacturers. An inspection of the separator bowl and the operating water device is made after six months or 4000 operating hours at the most. The lubrication oil should be changed every 4000 h, or once a year if the total number of operating hours is less than 4000 h/year. Finally, an overhaul of the complete separator system must be performed after 18 months or 12,000 operating hours at the most, where friction blocks, seals, bearings, and flat belt are renewed.

However, without knowledge of current device conditions, routine maintenance can result in premature activity, which may cause more problems than they fix [3].

Predictive maintenance sometime includes systematic machine monitoring to determine the mechanical conditions during operation, and to detect any problems quickly. In some ways, diagnostic technologies can predict the future, avoid unnecessary maintenance, and minimize irreversible failures. Therefore, the costs of such programs are minimized and compensated by other savings [3].

The risk of repeated failures in mechanical systems frequently leads to searches for the causes of the problem. For this reason, it is essential to implement new maintenance techniques in merchant marine, such as predictive maintenance based on vibration, acoustic emission, or motor current analysis to prevent failures or break-downs in the system.

In some pieces of equipment, such as separator systems for cleaning fuel and lube oils for diesel engines, which is a machine with rotating parts, there are signals suggesting high levels of vibration. Therefore, one of the ways of eradicating these risks is to recognize the symptoms by vibration. The most extended method in the industry is to measure vibration using accelerometers in specific points [24]. Generally, the study of vibrations has been focused on rolling elements [16,25,26] as fundamental sources of maintenance procedures. The main contribution of this paper will be to propose computational algorithms based on the vibrations of a mechanical system (separation systems), which allow for knowing the behavior and the state of the machine [27,28]. Vibration analysis makes it possible to extract the representative features of the whole device that could be related to the lifetime of their pieces, without determining whether a particular piece is failing, but indicating that a maintenance operation is required. The vibrations obtained were processed, and following this, they were used in a genetic neuro-fuzzy System in order to determine the relations between the dominant frequency and the working hours.

## 4. Experimental Study

**Figure 1:**
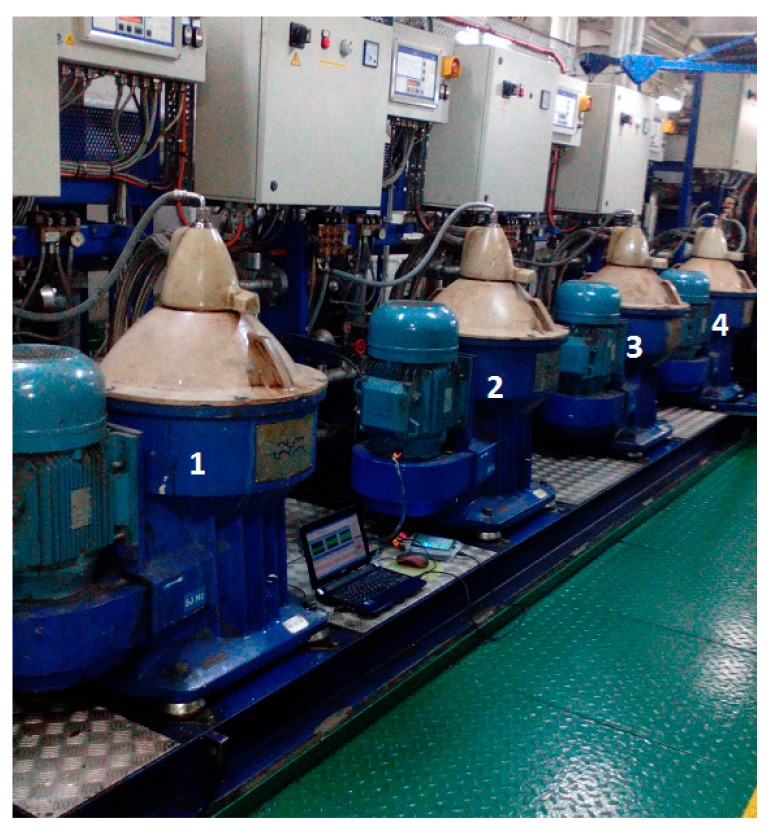
Photography of four marine oil Separators Alfa Laval SA 831 labeled with their corresponding numbers.

As was mentioned in previous sections, several actual measurements were carried out. Particularly, on board centrifugal separators of Ro-Pax vessels to transport passengers and freight have been considered. These vessels usually have a separation system that is formed by certain number of marine oil separations. In this case, Alfa Laval SA 831 separators systems, as shown in Figure 1, have been considered. The technical characteristics of this model are presented in Table 1.

Vibration analysis is used as the best tool for detecting changes in the behavior of rotating machinery, which is why a vibration study was carried out for each separation system. Vibration signals contain much information about the mechanical system [29]. For this reason, an accelerometer was attached to all oil separators, and five consecutive vibration measurements were made for 2 s with a sampling frequency of 2560 Hz.

Vibration signals were obtained through a miniature triaxial accelerometer sensor, Bruel & Kjaer 4504A (Bruel & Kjaer, Naerum, Denmark). This sensor has three independent outputs for simultaneous high-level measurements in three mutually perpendicular directions. The sensitivity of the accelerometer is 10 mV/g, and it measures a range of up to 9.0 KHz, and its lower bound of the sensing frequency range is 1 Hz. It is important to remark that each transducing element is individually calibrated.

**Figure 2:**
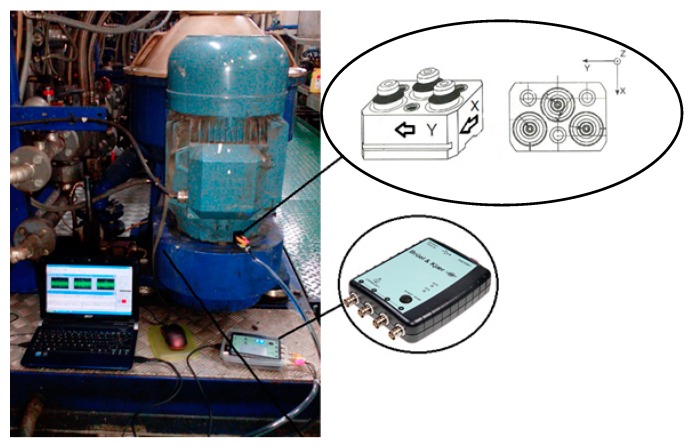
Assembly for measuring vibration over the separation system.

The accelerometer was connected to a Brüel & Kjaer PHOTON+ dynamic signal analyzer. The PHOTON+ is a versatile analyzer for sound and vibration measurements and recording, and for signal post-processing. It consists of a small data acquisition hardware unit and PC software. It allows for real-time signal analysis. In fact, it could be used as a FFT analyzer with a measurement dynamic range of 115 dB and an 84 kHz real-time rate. Figure 2 shows a photograph of the final assembly for vibration measurement on a marine separation system. It can be seen that the triaxial accelerometer attached to the oil separation system is linked to the analyzer, and this is finally connected to a laptop where the measurements were recorded [30]. The orientation of the accelerometer is as shown in Figure 2: the *X* axis is vertical, the *Y* axis goes through from right to left, and the *Z* axis traverses the device from front to back.

## 5. Intelligent Feature Extraction Method

Maintenance-controlling systems are one of the most important aspects of technical management, and they are considered to be one of the biggest challenges by ship managers [31]. The purpose of this work is to look for efficiency-based maintenance for the oil separators through intelligent condition monitoring. 

Currently, the maintenance procedure is simply based on the number of hours that the separator has been working. This kind of maintenance generates a great outlay to shipowners, owing to the fact that it is possible to take advantage of a device if it can run for longer. Furthermore, a maintenance operation can be necessary before a scheduled one because of a failing piece. For this reason, the number of working hours has been considered as a key parameter in this work. In this sense, it would be convenient to correlate this parameter in any way with the internal state of the system. Furthermore, the internal state has been studied in this paper, through the vibration signature.

Vibration measurements were done over real oil separators, as was exposed in the previous section. From those recorded vibrations, a signal processing stage was accomplished in order to extract their characteristic parameters. Specifically, FFT was applied. This process allows the frequency domain of the separator vibrations to be known. In particular, it the first five frequencies with a greater amplitude in the FFT spectrum were collected, along with their corresponding amplitudes in that domain as indirect parameters that were used to link the internal state of the separator and the number of working hours. In order to achieve this purpose, an intelligent method based on training was used, particularly a genetic neuro-fuzzy system [32,33,34], with a structure that was analogous to the one proposed by Jang [35]. The architecture of the three layers was utilized, as can be seen in [36]. Note that the genetic neuro-fuzzy inputs corresponded to the first layer, which represent the membership functions, and the outputs of this layer are expressed by Equation (1).

(1) $$P_{ij}=\exp\left(\frac{-{\left(U_{i}-m_{ij}\right)}^{2}}{\sigma_{ij}{}^{2}}\right)\begin{array}{c} j=1,2,\ldots ,N_{2}\\ i=1,2,\ldots ,N_{1} \end{array}$$

*N*_1_ represents the input number; in this paper there are 2; the frequency domain and its FFT amplitude. *N*_2_ is the number of nodes of the intermediate layer; thus, *U_i_* symbolizes the *i*-th input, *m_ij_* and *σ_ij_* the center and the width of the membership function, respectively, and finally, *P_ij_* is the output neuron with the *i*-th input and the output connected to the *j*-th node of the intermediate layer.

Equation (2) shows the output of the second layer of the genetic neuro-fuzzy, which is the rule system.

(2) $$\begin{array}{cc} \lambda_{i}=\min\left|p_{1j},p_{2j},\ldots ,p_{N_{1}j}\right| & j=1,\ldots ,N_{2} \end{array}$$

Finally the global output is given by Equation (3), where *N*_3_ is the genetic neuro-fuzzy output number. In this paper, *N*_3_ is 1, specifically the working hours corresponding to the state of the oil separator given by the input. The parameters *sv_jk_* are the estimated value of the *k*-th output given by the *j*-th node.

(3) $$\begin{array}{cc} Y_{k}=\frac{\sum_{j=1}^{N_{2}}s\nu_{jk}\lambda_{j}}{\sum_{j=1}^{N_{2}}\lambda_{j}} & k=1,\ldots ,N_{3} \end{array}$$

As Equations (1)–(3) show, the genetic neuro-fuzzy system depends on: the center (*m_ij_*) and width (*σ_ij_*) of the membership function, the estimated system outputs (*sv_jk_*), and the number of nodes of the intermediate layer (*N*_2_). A three-phase learning algorithm has been carried out in order to fix these parameters. The first two phases provide initial values and optimize the number of nodes of the hidden layer, that is, the number of rules. The parameters obtained previously are reset at the third phase.

### 5.1. Fist Level: Unsupervised Learning Phase

In this phase, the initial values to the center of the membership function (*m_ij_*) and the estimated system outputs (*sv_jk_*) are provided. For that, a Kohonen’s self-organizing [37] map is applied, and their inputs are:

(4) $$V=\left(U_{1}U_{2}\ldots U_{N_{1}}Y_{1}Y_{2}\ldots Y_{N_{3}}\right)$$

The vector (*U*_1_, *U*_2_,…, *U_N_*_1_) is the input vector to the genetic neuro-fuzzy system, and (*Y*_1_, *Y*_2_,…, *Y_N_*_3_) corresponds to the desired output vector.

The initial weight vector of the self-organizing map is acquired using the mean between the maximum and minimum of the input given by the user. Its dimension will be the input numbers (*N*_1_) plus output numbers (*N*_2_) as Equation (4) shows:

(5) $$W_{j}=\left(w_{1j}w_{2j}w_{3j}\ldots w_{N_{1}+N_{3}j}\right);\;j=1,2,\ldots ,N_{2}$$

A monodimensional Kohonen self-organizing map is applied in this work, in order to achieve the winner node. This new value allows for an update of the weights:

(6) $$m_{ij}=w_{ij};j=1,2,\ldots ,N_{2};i=1,2$$

This chosen learning algorithm is a typical unsupervised learning algorithm; therefore, once the algorithm has concluded and the winner node has been acquired, an assignment of the center of the membership functions (*m_ij_*) and the estimated system outputs (*sv_jk_*) will be carried out. As Equation (6) shows, the centers of the membership functions are obtained to *N*_2_, the first value of the *W_j_* vector, while the values for the estimated outputs are chosen using the rest of its components:

(7) $$sv_{jk}=w_{N_{1}+k,j};k=1,2;j=1,2,\ldots ,N_{2}$$

An adequate initial assignment of these values is essential, since they are a starting point. After that, in the following learning phases, these parameters will be updated from the initial ones.

### 5.2. Second Level: The Genetic Algorithm Phase

An optimization process is necessary with the purpose of obtaining a minimum number of rules. Since the number of nodes in the hidden layer is associated with the number of rules, this one should be optimized previously. In the unsupervised phase *N*_2_, *m_ij_*, and *sν_jk_* were obtained, but then there are still values for the parameters *σ_ij_* missing. For that, a genetic neuro-fuzzy system is built in the current phase, in order to establish adequate values for the parameter *sv_jk_*, and to accomplish a more reduced number of nodes in the hidden layer.

The genetic algorithm [32,33,38] developed in this phase employs the biological model of genetic evolution. On the one hand, there is an individual with basic information, and in this work a vector is established as the individual; on the other hand, elements known as genes can be found, and in this study, the vector components are the genes. In this way, the hidden nodes by a Boolean value and the width of the membership functions values are represented by the components of each vector. Therefore, each individual is expressed by the equation:

(8) $$V=\left(\begin{array}{ccc} 0\vee 1 & 0\vee 1 & 0\vee 1\ldots \\ \sigma_{21} & \sigma_{22}\ldots & \sigma_{2N_{2}}\ldots \end{array}\begin{array}{ccc} \sigma_{11} & \sigma_{12}\ldots & \sigma_{1N_{1}}\\ \sigma_{N_{1}1} & \sigma_{N_{1}2}\ldots & \sigma_{N_{1}N_{2}} \end{array}\right)$$

As it can be seen in Equation (8), the first N_2_ element values can be 1 or 0, depending on whether the corresponding rule is taken into account or not, respectively. The last elements of the vector correspond to the membership functions values.

A fitness function is defined, considering the difference between the real output values and the individual output values. It is defined as the mean squared error (MSE) and it is expressed by Equation (9). Owing to each vector is a possible trained system, and the values obtained in Equations (6) and (7) are taken for all individuals in this learning phase, after the genetic neuro-fuzzy algorithm is applied, individual satisfactory values for *σ_ij_*, and an adequate set of *N*_2_ rules (nodes on the hidden layer) can be obtained:

(9) $$F=\frac{1}{2}\sum_{j=1}^{N_{2}}{\left(Y_{k}-S_{k}\right)}^{2}$$

### 5.3. Third Level: Supervised Learning Phase

After obtaining initial values for the *m_ij_*, *σ_ij_*, and *sv_jk_* parameters of the genetic neuro-fuzzy system, the last phase attempts to improve these values. The system used in this work is quite similar to a three layer neural network; therefore, the standard learning algorithms adapted to the mathematical expressions of these particular nodes can be applied. The same mathematical expression as the neurons in a radial basis neural network [39] has been used to express the nodes on the input layer of the genetic neuro-fuzzy system; moreover, the least mean squared learning algorithm has been also applied. After this process, it is intended to minimize the criterion function, which is defined as the error function between the outputs of the genetic neuro-fuzzy system (*S_k_*) and the real outputs (*Y_k_*), as shown by Equation (10):

(10) $$E=\frac{1}{2}\sum_{k=1}^{N_{3}}{\left(Y_{k}-S_{k}\right)}^{2}$$

## 6. Results and Discussion

**Figure 3:**
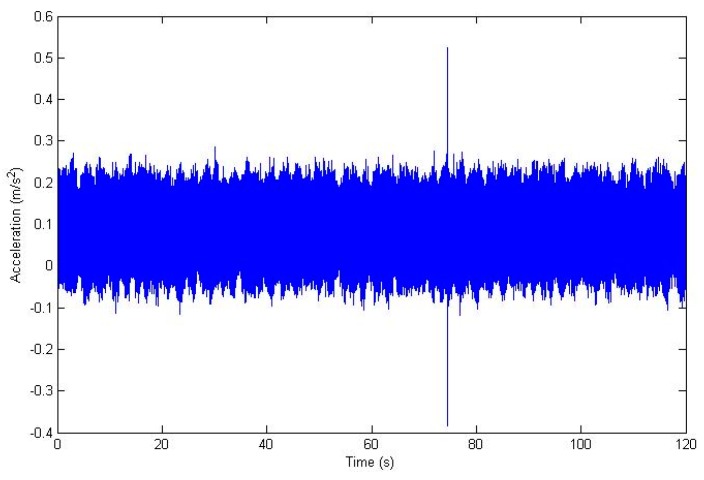
The vibration signal on the *X* axis measured in oil separator system number 2.

**Figure 4:**
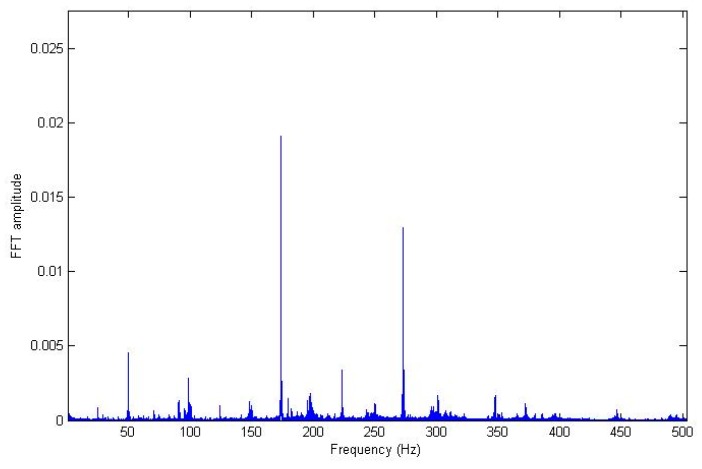
The frequency domain of vibration signal on the *X* axis measured in oil separator system number 2.

Before starting the training, a processing signal was necessary to extract the minimum information about the state of the system as an input of the neuro-fuzzy system. The data collected in the vessel were vibration signals, and with this intelligent method, a vibration signature was intended to be related with the corresponding working hours for which the oil separator system was running. For that reason, the vibration measurement was processed by a traditional FFT, in order to extract the relevant information about frequency domain. Particularly, the five dominant frequencies were obtained in each measurement. It was decided that these dominant frequencies would be used with their corresponding amplitude FFTs as the input vectors to the genetic neuro-fuzzy system. Therefore, the output is the number of working hours of the oil separator from which that vibration signal was obtained. In other words, once a vibration signature has been measured, the trained system will be able to provide the number of hours that it had been working. It is important to highlight the fact that every measurement featuring in this study was carried out in real environments; that is, in actual vessels in the middle of actual journeys. Figure 3 shows an example of a signal before it is processed. This graphics correspond to a vibration measured on one of the oil separation systems, particularly on the system labeled as number 2, and on the *X* axis. Figure 4 displays the frequency spectrum of signal shown in Figure 3.

Once the collected signals were processed, the input data set was ready to be introduced in the proposed AI method. Each input vector was composed using the five dominant frequencies and their corresponding amplitudes for each packet of two second signals. Following, each input vector was fixed with an output value, which was the number of working hours that this particular oil separator system had been running until the moment of measurement.

**Figure 5:**
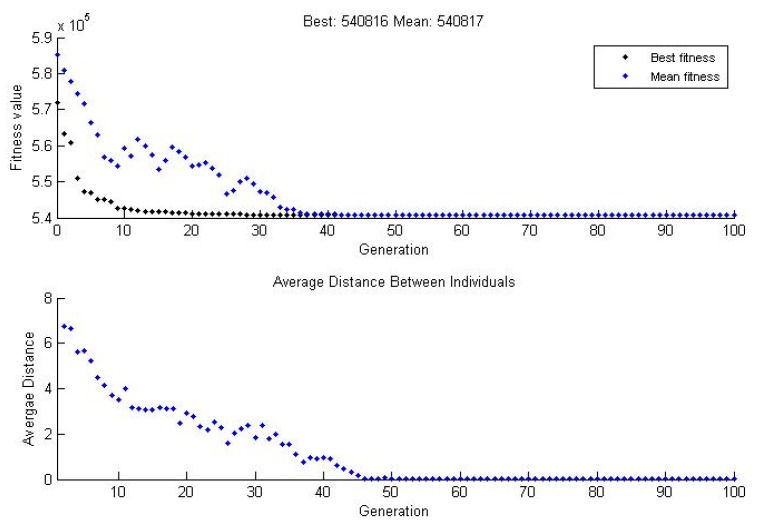
Evolution of the fitness value and the average distances between individuals.

With the input–output data set in each training phase previously explained, several trials were carried out in order to obtain the parameters that provide an adequate error value. It is important to remark that 70% of the data was used to train the system, while the other 30% was reserved to test the generalization capability of the trained algorithm. The genetic neuro-fuzzy system can be said to reach a suitable level of generalization if it provides adequate outputs to unknown input values, meaning the data that had not been used in the training process. After the already-mentioned trials, one with a minimum error function was chosen. Figure 5 shows the result of the genetic algorithm phase. As it can be seen, after 35 generations, the best fitness value was quite similar to the mean, and after 45 generations, the average value between individuals was zero; therefore, the algorithm achieved good training. Moreover, if more particular results were analyzed, satisfactory parameters were obtained as well. Particularly, the genetic neuro-fuzzy system reached a training error of 1439.3093 h, and a generalization error of 1189.2021 h. At this point it is necessary to highlight that the on board oil separator systems used for this study had between 2000 and 7000 working hours; therefore, the error value achieved by the algorithm was reasonably adequate. 

Finally, in order to ensure that the genetic algorithm reached a suitable generalization level, Table 2 is presented. It shows a comparison between the real value of the working hours that the particular oil separator system had in the moment of measurement, and the value provided by the genetic neuro fuzzy system once it was trained. It is important to point out that the input values are the data set reserved to test the generalization capability, which means that they are unknown for the system. As can be seen in Table 2, the error was about 1500 h or less. This value was reasonable since the maximum working hours is 12,000, and even if the system indicated a working hour number above or below 1500, it was still inside the interval under consideration. These intervals are considered taking into account the proximity to an overhaul. This means that when the number of working hours was below 5000, the oil separator system had been running for a short amount of time since the last overhaul, whereas if it was near 7000, an overhaul was due quite close, and if it was above to 10,000, the overhaul was imminent.

## 7. Conclusions

The developed work presents condition-based maintenance for oil separator systems through a genetic neuro-fuzzy system. Actual measurements of vibrations were carried out on these systems on Ro-Pax vessels in order to link these signals with the internal state of the separation systems. Following this, a signal processing stage with a fast Fourier transform was applied, in order to extract the frequency domains of the separator vibrations. Particularly, the first five frequencies with greater amplitude in the FFT spectrum with their corresponding amplitudes were used as input data set for the training process. Each input vector was fixed with the number of working hours that this particular oil separator system had been running for until the measurement moment.

After the training process, it can be concluded that the vibration signature of an oil separator system provides information capable of preventing damages. This signal is related to its internal state, and an artificial intelligence technique can indicate the number of working hours that the system have been running for. The proposed method presents an improvement over the preventive scheduled maintenance that is carried out when a certain number of hours has been achieved, and this technique can indicate whether it is possible to extend the next maintenance service if the system is healthy, or if it is necessary to perform maintenance ahead of time if the separator system shows failure indications. This capability could turn out to be attractive to shipowners, due to the fact that it can prevent breaks or delay a revision, and therefore, it would imply an economic advantage.

## Figures and Tables

**Table 1:** Marine oil separator characteristics.

Characteristic	Value
Electrical current frequency	50 Hz
Motor power (50 Hz)	7.5 kW
Motor speed synchronous (50 Hz)	3000 rpm
Bowl speed, synchronous (50 Hz)	10.683 rpm
Maximum density of feed/sediment	1100/2659 kg/m^3^
Maximum density of operating liquid	1000 kg/m^3^
Feed temperature, min./max.	0 °C to 100 °C
Maximum viscosity of operating liquid	700 cSt at 50 °C

**Table 2:** Comparison between outputs provided by the genetic neuro-fuzzy system, and the real values of the working hours.

Real Working Hours	Working Hours Provided by Genetic Neuro Fuzzy
2885	4339
3090	4338
5887	4337
6300	6411
3078	4231
3248	4570
5621	4725
6476	4996
